# Multifaceted Functions of Platelets in Cancer: From Tumorigenesis to Liquid Biopsy Tool and Drug Delivery System

**DOI:** 10.3390/ijms21249585

**Published:** 2020-12-16

**Authors:** Melania Dovizio, Patrizia Ballerini, Rosa Fullone, Stefania Tacconelli, Annalisa Contursi, Paola Patrignani

**Affiliations:** 1 Department of Neuroscience, Imaging and Clinical Science, “G. d’Annunzio” University, 66100 Chieti, Italy; m.dovizio@unich.it (M.D.); rosa.fullone@unich.it (R.F.); s.tacconelli@unich.it (S.T.); annalisa.contursi@unich.it (A.C.); 2 Center for Advanced Studies and Technology (CAST), 66100 Chieti, Italy; patrizia.ballerini@unich.it; 3 Department of Innovative Technologies in Medicine and Dentistry, “G. d’Annunzio” University, 66100 Chieti, Italy

**Keywords:** platelets, cancer development, tumor-educated platelets, platelet-derived molecules, drug delivery system, extracellular vesicles

## Abstract

Platelets contribute to several types of cancer through plenty of mechanisms. Upon activation, platelets release many molecules, including growth and angiogenic factors, lipids, and extracellular vesicles, and activate numerous cell types, including vascular and immune cells, fibroblasts, and cancer cells. Hence, platelets are a crucial component of cell–cell communication. In particular, their interaction with cancer cells can enhance their malignancy and facilitate the invasion and colonization of distant organs. These findings suggest the use of antiplatelet agents to restrain cancer development and progression. Another peculiarity of platelets is their capability to uptake proteins and transcripts from the circulation. Thus, cancer-patient platelets show specific proteomic and transcriptomic expression patterns, a phenomenon called tumor-educated platelets (TEP). The transcriptomic/proteomic profile of platelets can provide information for the early detection of cancer and disease monitoring. Platelet ability to interact with tumor cells and transfer their molecular cargo has been exploited to design platelet-mediated drug delivery systems to enhance the efficacy and reduce toxicity often associated with traditional chemotherapy. Platelets are extraordinary cells with many functions whose exploitation will improve cancer diagnosis and treatment.

## 1. Introduction

Platelets are known for their role in hemostasis and thrombosis [1,2]. They are anucleated cells arising from cytoplasmic fragmentation of megakaryocytes, mainly in the bone marrow, and contain various mediators stored in at least three major types of granules—α-granules, dense granules, and lysosomes. Platelets represent an essential link between tissue damage/dysfunction and inflammatory response, acting to repair the injury; however, if platelet activation is uncontrolled, it leads to chronic inflammation associated with many pathological conditions, including atherothrombosis [3] and cancer [4,5]. The analysis of randomized clinical trials (RCTs) with aspirin, performed to evaluate cardiovascular benefits has shown that daily administration, even at low doses (75–100 mg), is associated with a reduced incidence of colorectal cancer (CRC) and long-term mortality [6,7]. Low-dose aspirin can interfere with the molecular mechanisms involved in early-onset CRC [8]. 

Aspirin is responsible for cardiovascular protection at low doses through preferential inhibition of platelet cyclooxygenase (COX)-1 activity [9,10]. The extent of inhibition of platelet COX-1-dependent thromboxane (TX)A_2_ generation and the clinical benefit are saturable at daily low doses of aspirin [9,10,11,12]. Since platelets do not have a nucleus, the irreversible inhibitory effect of aspirin toward platelet COX-1 persists for the lifespan of the platelet (between 5 and 10 days) [9], even though the drug exhibits a short half-life (approximately 20 min) [10,11]. Irreversible enzyme inactivation in an anucleated cell with a long lifespan in vivo explains the virtually complete inhibition of platelet COX-1 activity (>97%) by low-dose aspirin given once a day [9,10,11]. In contrast, in a nucleated cell, the aspirin-irreversible inhibition of COX activity is rapidly recovered through de novo protein synthesis; thus, multiple daily dosing is necessary to obtain an adequate inhibitory effect of prostanoid generation, translating into therapeutic effects [10]. Patrignani et al. [13] have shown that the aspirin-inhibitory effect on colon cancer cells constitutively expressing COX-2 is completely recovered 24 h after drug exposure. Prostaglandin (PG)E_2_, the main product of COX-2 activity in epithelial and stromal cells, modulates apoptosis, cell proliferation, and migration [14]. It is unlikely that low-dose aspirin given once a day exerts anticancer effects by directly inhibiting COX-2-dependent PGE_2_ biosynthesis in nucleated cells [13]. Low-dose aspirin can affect COX-2 expression in stromal cells and cancer cells by inhibiting platelet activation and the release of several molecules, such as growth factors, cytokines, and lipids, including TXA_2_, which contribute to COX-2 induction in the tumor microenvironment and cancer cells [8,15,16,17]. All these lines of evidence support the pivotal role of platelets in promoting early cancerogenic signaling pathways restrained by aspirin [8,15,16]. 

The impact of low-dose aspirin on COX-2 activity in vivo in humans has been studied by assessing indices of the systemic biosynthesis of PGE_2_ and PGI_2_ (prostacyclin), i.e., the urinary levels of PGE-M (11-α-hydroxy-9, 15-dioxo-2,3,4,5-tetranor-prostane-1, 20-dioic acid) [18,19] and those of PGI-M (2,3-dinor-6-keto-PGF_1α_) [20], respectively. They are the major urinary enzymatic metabolites of the parent prostanoids. In 40 individuals undergoing CRC screening treated with enteric-coated low-dose aspirin (100 mg daily for a week), Patrignani et al. [13] found a significant (*p* < 0.05) small reduction (13%) in the median urinary values of PGE-M. Urinary PGI-M levels did not differ in a statistically significant fashion [13]. These may data suggest a marginal inhibitory effect by a low dose on indices of COX-2 activity in vivo. However, Boutaud et al. [21] reported that low-dose aspirin (81 mg daily) reduced urinary PGE-M and PGI-M levels by 45 and 37%, respectively, in a population of 52 individuals with a median age of 68 years and of whom 50% were current or former smokers. It is noteworthy that the selective COX-2 inhibitor celecoxib (200 mg of BID) reduced median urinary PGE-M levels by approximately 54% in healthy volunteers, never smokers, or current smokers [19]. The possible contribution of COX-1 to the urinary levels of PGE-M cannot be excluded, at least in some clinical conditions associated with the activation of platelets, which generate PGE_2_ as a minor product of arachidonic acid (AA) metabolism. In healthy volunteers, single dosing of 400 mg of celecoxib significantly reduced average urinary PGI-M levels by 70–80%, which was not significantly different from the inhibitory effect of the nonselective nonsteroidal anti-inflammatory drugs (NSAID) ibuprofen (800 mg) [20]. These results suggest a dominant contribution of COX-2 to the systemic biosynthesis of prostacyclin [22]. The assessment of urinary levels of PGE-M and PGI-M does not indicate the cellular source or COX-isozyme of altered prostanoid generation, particularly in pathological conditions. To obtain definitive information on the impact of low-dose aspirin on COX-2 expressed in cancer lesions, it is necessary to use a direct biomarker of drug action, such as the assessment of the extent of acetylation of COX-2 at serine 516, which has been recently developed by Tacconelli et al. [23].

Nonsteroidal anti-inflammatory drugs (NSAIDs), including aspirin, can inhibit the proliferation and induce the apoptosis of colon cancer cells in vitro independently from their effects on COX-dependent prostanoid biosynthesis (reviewed in [24]). However, it is unlikely that low-dose aspirin can act as an anticancer agent via these mechanisms because they occur at millimolar concentrations. It is noteworthy that aspirin’s maximal systemic plasma concentration (Cmax) after dosing with 100 mg of enteric-coated aspirin is approximately 4 μM [11].

The use of low-dose aspirin is recommended in CRC prevention strategies [25]. Furthermore, a crucial challenge remains in identifying cancer patients who would have a more significant benefit from aspirin therapy. This would avoid the exposure of unresponsive patients to aspirin side effects, including the risk of severe bleeding, such as the bleeding of blood vessels in the brain or gut [8,26,27].

Platelets contain a changing repertoire of proteins and genetic material, including transcripts and microRNAs [28], i.e., small noncoding RNAs, that act as regulators of gene expression by posttranscriptional mechanisms [29]. Upon platelet activation, biologically active molecules are secreted in a soluble form or packaged into extracellular vesicles (EVs) [30,31]. These mediators promote chronic inflammation implicated in different precancerous lesions [32]. 

Numerous experimental findings indicate that platelets contribute to tumor cell extravasation and metastasis [4,5]. The results of daily aspirin RCTs have shown a reduced distant metastasis frequency in patients who developed different types of cancer [33,34,35]. Interestingly, a 75 mg controlled-release preparation of aspirin with low bioavailability, which inhibits platelet COX-1 in the presystemic circulation associated with marginal systemic effects [36], was used in the Thrombosis Prevention Trial (TPT) for the primary prevention of cardiovascular disease [37]. This trial was included in Rothwell’s analysis of five large randomized trials of daily aspirin (≥75 mg daily) versus control [33]. The results of TPT showed that aspirin reduced the risk of distant metastasis by 30–40% and reduced the risk of metastatic adenocarcinoma by almost half [33]. Altogether, the evidence obtained in experimental and clinical studies sustains antiplatelet agent benefit to restrain cancer cells from spreading to distant organs. 

The activation of the coagulation system is associated with unfavorable patient survival. This issue is discussed in many excellent papers [4,38] and is not included in the present review.

Another peculiarity of platelets is that they uptake circulating proteins and RNAs/microRNAs, thus acquiring a different intracellular molecular repertoire specific to the individual clinical condition. The analysis of platelet content, as a whole of transcripts (transcriptomics: the analysis of mRNAs and microRNAs) or proteins (proteomics), has the promise of being a novel tool for the diagnosis and prognosis of several diseases, including cancers (known as the liquid biopsy) [39,40]. 

Platelets can also be appropriate as drug delivery systems since, upon activation, they release vesicles that deliver their content to other cells, including cancer cells. Loaded platelets with an anticancer agent administered to mouse models of cancer deliver the compound to malignant cells via the release of vesicles containing it [41].

## 2. Role of Platelets in the Early Events of Tumorigenesis

Platelets are activated in response to altered functions of vascular cells and intestinal mucosal cells, possibly dependent on lifestyle and aging [8,15,16]. Activated platelets release several lipid mediators, including the products of arachidonic acid metabolism (i.e., mainly TXA_2_ and PGE_2_), a-granule proteins (both angiogenic and antiangiogenic factors, growth factors, proteases, and many cytokines), and different types of vesicles rich in micro–ribonucleic acids (microRNAs) [8,16]. Due to this capacity, activated platelets trigger numerous signaling pathways in different cell types, such as vascular, immune, epithelial, tumor cells, and fibroblasts [42]. In colonic mucosa, these events induce a tissue microenvironment, promoting intestinal neoplasia [8]. Enhanced levels of TXA_2_ activate fibroblasts, which acquire proliferative and migratory properties [43]. Moreover, platelet-derived molecules can induce angiogenesis [44]. We recently generated a mouse with specific deletion of COX-1 in platelets that allowed us to identify TXA_2_-dependent platelet activation as a central mechanism in intestinal inflammation and fibrosis in vivo [43].

A key event in intestinal tumorigenesis is represented by the enhanced biosynthesis of PGE_2_ [45]. It can occur early in epithelial cells via COX-1 activity, associated with the repression of the prostaglandin-degrading enzyme 15-prostaglandin dehydrogenase (15-PGDH) expression [46,47]. Mucosal PGE_2_ can phosphorylate ribosomal protein S6 (p-S6) via PKA [48]. S6 is a crucial regulator of the 40S ribosome biogenesis transcriptional program, and its phosphorylation is closely related to cell growth capacity and tumor progression [49]. We have found that the rectal mucosal p-S6/S6 ratio significantly correlated with PGE_2_ levels [13]. Interestingly, we have shown that the administration of enteric-coated low-dose aspirin (100 mg daily) to individuals undergoing CRC screening can cause long-lasting acetylation of COX-1 and reduced phosphorylation of S6 in apparently healthy colorectal mucosa [13]. This effect may interfere with early colorectal carcinogenesis. Low-dose aspirin causes a significant inhibitory effect on platelet COX-1, and a lower inhibition of COX-1 expressed in colorectal mucosa associated with rectal mucosa changes phenotype [13].

A further increase of PGE_2_ production occurs at later stages of tumorigenesis associated with induction of COX-2 and the downstream synthase, microsomal PGE_2_ synthase-1 (mPGES-1; a major terminal PGE_2_ synthase), in stromal cells and then in epithelial cells [50,51]. Enhanced PGE_2_ production alters the normal apoptotic processes involved in preventing the accumulation of genetic mutations and leads ultimately to enhanced proliferation [45,52]. Moreover, PGE_2_ plays a role in suppressing immune functions and facilitating tumor immune escape [53]. 

Platelet-derived mediators also induce epithelial–mesenchymal transition (EMT) programs, where polarized, immobile epithelial cells become migratory mesenchymal cells, promoting tumorigenesis and facilitating metastatic colonization [54].

All these findings sustained the benefit of low-dose aspirin, and possibly other antiplatelet agents, in CRC prevention. Inhibition of platelet function can restrain the cascade of molecular and biological events associated with tumorigenesis occurring in the stromal compartment and epithelial cells.

## 3. Platelets and T Cell Immunity

Platelets can directly depress T cell function both in vitro and in vivo [55]. Upon activation, platelets release transforming growth factor (TGF)-β, which exerts an immunosuppressive effect. Moreover, platelets express TGF-β-docking receptor glycoprotein A repetitions predominant (GARP), which acts by increasing the activation of latent TGF-β [55]. Specific deletion of GARP in platelets mitigated TGFβ activity at the tumor site and potentiated protective immunity against both melanoma and colon cancer [55]. T cell therapy of B16 melanoma was improved by concurrent treatment with aspirin and clopidogrel (an antiplatelet agent acting as P2Y12 antagonist) [55], suggesting that platelets can suppress antitumor T cell immunity.

Natural killer (NK) cells are cytotoxic lymphocytes that play an essential role in tumor immunosurveillance, preferentially eliminating targets with a low or absent expression of MHC class I and stress-induced expression ligands for activating NK receptors [56]. It has been shown that platelets promote metastasis by protecting disseminating tumor cells from NK cell immunosurveillance [57]. Tumor cells rapidly coated in the presence of platelets in vitro and circulating tumor cells of cancer patients display coexpression of platelet markers [58]. The transfer of MHC class I onto the tumor cell surface results in high expression levels of platelet-derived normal MHC class I [58]. The resulting “phenotype of false pretenses” disrupts the recognition of the tumor cell missing self [59], thereby impairing cytotoxicity and IFN-γ production by NK cells [58]. This is a mechanism by which malignant cells may utilize platelets to impair NK cell ability to prevent metastasis. Improved knowledge of platelet role in immune evasion of tumor cells will open the way to develop new antimetastatic therapies.

## 4. Role of Platelets in Metastasis

Platelet–tumor cell interactions in the circulation contribute to the metastatic dissemination of epithelial tumors [4]. The formation of platelet aggregates surrounding tumor cells can support tumor cell survival and protection from immune elimination [4]. Moreover, platelets contribute to tumor cell adhesion to the endothelium, which translates to tumor cell arrest and extravasation [4].

Platelets and cancer cells interact reciprocally. Platelets directly associate with cancer cells using different molecules depending on specific receptors expressed in cancer cells [5,16] (Table 1). Thus, distinct pharmacological agents are necessary to interrupt the direct crosstalk between platelets and different cancer types. In Table 1, the known determinants of the interactions between platelets and cancer cells are reported with possible inhibitory pharmacological tools that have been described. These findings suggest that selecting a specific pharmacological agent to constrain metastasis development requires the characterization of the altered expression of tumor molecules involved in the interaction with platelets.

Events associated with cancer-dependent platelet activation is the release of TXA_2_ and PGE_2_ (as a minor product) and proteins from a-granules [such as TGF-β and platelet-derived growth factor (PDGF)], which contribute to tumor cell dissemination into the bloodstream [16].

Labelle et al. [73] showed that direct platelet interaction with tumor cells synergizes with platelet-released TGF-β to induce EMT, therefore increasing tumor cell invasive potential and their capacity to colonize the lung.

Dovizio et al. [17] found that PDGF released by platelet/colon cancer cell interaction contributes to the COX-2 induction in tumor cells via a posttranscriptional mechanism. Moreover, the interaction of platelets with cancer cells induces changes in the mRNA levels of EMT markers, thus conferring greater migratory and metastatic capacities [74]. Platelets can adhere to the human colon adenocarcinoma cell line HT29 through the interaction of the platelet collagen receptor GPVI with galectin-3, which is highly expressed in tumor cells and contains a collagen-like domain [17]. The role of platelet GPVI was elucidated using Revacept, a fusion protein containing the extracellular domain of human GPVI [63], which binds specifically to collagen at the vascular injury site, thus inhibiting adhesion and platelet aggregation [75]. Revacept prevented platelet-induced changes in cancer cells, such as EMT-related genes and COX-2 expression [75].

Furthermore, Guillem-Llobat et al. [74] showed that the mesenchymal phenotype induced in colon cancer cells by exposure to platelets in vitro is characterized by enhanced prothrombotic and metastatic properties when injected in vivo into mice. These effects were mitigated by administering low-dose aspirin to mice, suggesting a pivotal role of platelet activation in metastasis development [74].

Platelet-–HT29 cell interaction causes the release of PGE_2_ from platelets [74], which promotes EMT and migration via the activation of the PGE_2_ receptor subtype EP4 on cancer cells [74]. These effects were prevented by different antiplatelet agents: aspirin (a selective inhibitor of platelet COX-1), DG-041 (an antagonist of the EP3 receptor), and ticagrelor (a P2Y12 receptor antagonist) [74].

Lucotti et al. [76] found that aspirin reduced lung metastasis through inhibition of platelet COX-1. The platelet COX-1/TXA_2_ pathway was responsible for this antimetastatic effect. Its inhibition affected platelet aggregation on tumor cells, endothelial activation, tumor cell adhesion to the endothelium, and the recruitment of metastasis-promoting monocytes/macrophages, diminishing the formation of a premetastatic niche [76].

## 5. Platelet-Derived Vesicles and Their Implication to Cancer

Platelets release EVs, a heterogeneous population of membrane-enclosed vesicles characterized by different sizes. Medium-size (100–1000 nm) EVs are also called microparticles (MPs) [77]. Platelet-derived MPs are shed from plasma membranes during platelet activation, stress, apoptosis, and necrosis, and they represent the most abundant population, accounting for 70–90% among circulating EVs in the peripheral blood of healthy people [78,79]. MP generation requires the loss of the platelet membrane phospholipid asymmetry by the enzymatic pathway “flip-flop” and scramblase that, lastly, results in the phospholipid rearrangement between inner and outer leaflets Notably, the exposure of anionic phosphatidylserine (PS) on the outer cell membrane of platelets is key to regulating thrombin generation [80].

Membrane-derived EVs are involved in cell–cell communication through different mechanisms: (i) directly stimulate cells as a kind of “signaling complex,” (ii) transfer membrane receptors, proteins, mRNA, and organelles (e.g., mitochondria) between cells, and, finally, (iii) deliver infectious agents into cells (e.g., human immunodeficiency virus, prions) (reviewed in [81]).

Platelet-derived MPs can transfer specific microRNAs to cancer cells in vitro and in vivo, thus promoting phenotypic changes [82,83].

Platelet-derived MPs can influence target cells via transcellular lipid metabolism, i.e., transferring AA to endothelial cells to induce the expression of COX-2, which, in turn, metabolizes AA to produce prostanoids [84].

Several studies focused on exploring the roles of EV role effectors in hemostasis, thrombosis, angiogenesis, immunity, inflammation, and cancer. In a recent study, higher circulating plasma levels of MPs were observed in patients with gastric cancer than in healthy controls and, more interestingly, plasma levels of MPs, along with interleukin (IL)-6, vascular endothelial growth factor (VEGF), and RANTES, were the highest in the patients with more advanced stages of the disease [85]. These observations reveal a role of platelet MPs in tumorigenesis and also suggest the usefulness of evaluating their count and phenotype to identify metastatic development in patients with gastric cancer [85].

A study conducted in obese individuals has shown that platelet-derived MPs of obese women showed higher heterogeneity in size and contained different levels of proteins relevant to thrombosis and tumorigenesis [86]. MPs from obese individuals presented the enhanced capacity to induce EMT and endothelial-to-mesenchymal transition (EndMT) in cancer cells and endothelial cells, respectively [86]. These effects might contribute to the increased risk of developing thrombosis and multiple malignancies observed in obesity [86].

The analysis of the proteomic cargo of plasma EVs in cancer patients recently identified a panel of tumor-specific proteins able to detect cancer and classify its primary origin, thus proponing the analysis of EV as promising for cancer detection [87].

## 6. Antiplatelet Drugs and Cancer

Results of clinical and experimental studies support that targeting platelet activation is involved in cancer development and the promotion of metastasis, opening the way to the possible chemopreventive use of antiplatelet agents [4,8,10,15,16]. The finding that low-dose aspirin (75–100 mg), which mainly targets platelets [9,10,11], reduces the incidence and mortality of several types of cancer, including CRC [6,7,8,33,34,35], led to its recommendation in the primary prevention of cancer [25]. The US Preventive Services Task Force stated that low-dose aspirin should be used for the primary prevention of cardiovascular disease (CVD) and CRC in adults aged 50–59 years “who have a 10% or greater 10-year CVD risk, are not at increased risk for bleeding, have a life expectancy of at least ten years, and are willing to take low-dose aspirin daily for at least ten years” [25]. However, we believe that some issues remain to be clarified before giving a conclusive recommendation on the use of aspirin for cancer chemoprevention. As discussed above, the knowledge of low-dose aspirin pharmacodynamics and pharmacokinetics supports that the drug acts as an anticancer agent by targeting platelet-dependent signaling pathways of tumorigenesis [8,9,10,11,13,15,16,17]. Low-dose aspirin can indirectly restrain COX-2 induction in an adenomatous lesion by inhibiting platelet function. Clinical studies in CRC patients treated with low-dose aspirin are undergoing to obtain conclusive evidence of the proposed mechanism. To this aim, we recently developed a direct biomarker of aspirin action by assessing the extent of acetylation of COX-2 in tissue biopsies [23]. The use of this novel biomarker of aspirin action will also be crucial to clarify whether higher doses of aspirin might translate into enhanced inhibition of COX-2-dependent PGE_2_ biosynthesis in cancerous lesions. It is also necessary to clarify whether (i) other antiplatelet agents exert anticancer effects and (ii) their coadministration can improve the chemopreventive effect of low-dose aspirin.

### 6.1. Aspirin

Aspirin, acetylsalicylic acid (ASA), is a member of NSAIDs. Unlike other NSAIDs, aspirin inhibits prostanoid biosynthesis by the irreversible inactivation of COX-1 and COX-2 through the acetylation of a specific serine residue located in the cyclooxygenase active site, at position 529 and 516 of COX-1 and COX-2, respectively (reviewed in [8,10,16]). Despite the short half-life (i.e., 20 min) of the drug, aspirin administration at low doses once a day causes an antiplatelet effect because of irreversible COX-1 inactivation (occurring both in the presystemic and systemic circulation) in the anucleated platelets characterized by a low rate of protein synthesis. Chronic administration with low-dose aspirin causes a virtually complete inhibition of platelet COX-1 activity (97%), maximal platelet acetylation of COX-1 (75%), and inhibition of platelet function persisting throughout the dosing interval (i.e., 24 h) [9,11].

The first evidence of aspirin’s chemopreventive effect against CRC was obtained in epidemiological studies. Both case–control and cohort studies have shown that the regular and continued use of aspirin is associated with an approximately 50% reduction in the incidence and mortality of CRC [88,89]. However, this benefit may not be evident until after at least a decade of regular aspirin consumption [90]. RCTs with different aspirin doses (81–600 mg daily) were carried out in different clinical conditions (Table 2) [91,92,93,94,95,96,97,98,99,100,101,102,103,104]. In two RCTs performed in healthy individuals (Physicians’ Health (PHS) and Women’s Health (WHS) Studies), aspirin (325 and 100 mg, respectively) failed to reduce the risk of CRC (Table 2) [91,92], possibly due to the inadequate treatment duration or follow-up and/or administration schedule (Table 2). Differently, in prospective, placebo-controlled RCTs performed in an average/high-risk population, aspirin at low–medium doses (81–325 mg daily) significantly reduced the risk of colorectal adenoma recurrence [93,94,95,96,97] (Table 2). Limited information is currently available on aspirin’s chemopreventive effect in familial adenomatous polyposis (FAP) patients. In the study performed by Burn et al. [98], an international, multicenter, randomized, placebo-controlled trial of aspirin (600 mg/d) and/or resistant starch (RS) (30 g/d) (CAPP1) performed in young FAP patients (from 10 to 21 years of age), the daily administration of 600 mg of aspirin from 1 to 12 years did not meet the primary end-point consisting of the reduction of the polyp number in the rectum and sigmoid colon. More recently, it was found that the same aspirin treatment, administered to Lynch syndrome patients (CAPP2 trial), was associated with a significant reduction of CRC development [99]. The results of the 10-year follow-up of the CAPP2 trial were recently published [88]. The data show that adult carriers of a pathogenic mismatch repair gene defect (Lynch syndrome) should take 600 mg aspirin daily for at least two years to significantly reduce future cancer risk. However, this effect does not become apparent for at least four years. Importantly, adverse events during the intervention phase between aspirin and placebo groups were similar [100]. The ongoing CaPP3 study aims to define the optimal dose of aspirin (600 mg daily versus 300 mg and 100 mg daily) for cancer prevention versus adverse events in Lynch syndrome.

Low-dose aspirin on cancer incidence and mortality was recently investigated in diabetic patients and elderly populations [101,102,103,104]. As reported in Table 2, Okada et al. showed data in type 2 diabetic patients [101] administered with aspirin at doses of 81 or 100 mg daily for 10.7 years, in which the drug caused a significant reduction of cancer incidence. Differently, the results of the A Study of Cardiovascular Events in Diabetes (ASCEND) trial showed that the rate of cancer-related mortality in type 2 diabetics patients was unaffected by low-dose aspirin administration [102]. In the elderly population administered with 100 mg/die of aspirin, the drug failed to satisfy the secondary end-point, i.e., cancer mortality rate [103,104]. All these studies have the limitation of a small cohort, which is inadequate to evaluate aspirin’s chemopreventive effect.

The ADD-Aspirin trial is an ongoing adjuvant trial in patients with newly diagnosed cancers, including colorectal, gastroesophageal, breast, and prostate cancer, administered with 100 mg or 300 mg/die of aspirin [105]. The results of this trial will clarify whether regular aspirin use after treatment for early-stage cancer can prevent cancer recurrence and death. Moreover, it will define the appropriate dose of aspirin to be administered (Table 2).

### 6.2. P2Y12 Receptor Antagonists

ADP, released from dense granules of platelets, induces platelet aggregation and is also involved in platelet granule secretion [2]. These effects occur via the activation of the three types of purinergic receptors expressed in platelets, the P2X-type ion channel-linked receptor, and two P2Y-type G protein-coupled receptors, P2Y1 and P2Y12 [106]. P2Y1 and P2Y12 are coupled to Gq and Gi proteins, which activate PLC and inhibit adenylyl cyclase, respectively [106].

ADP-dependent activation of the P2Y1 receptor leads to an increase in intracellular calcium via Gq coupling. P2Y12 activation translates into Gi coupling, thus resulting in the inhibition of adenylyl cyclase and the prevention of the phosphorylation of vasodilator-stimulated phosphoprotein (VASP), whose phosphorylation correlates with the inhibition of platelet function [107]. The stimulation of P2Y12 leads to the activation of phosphoinositide 3-kinase (PI3K) and Akt kinase [106].

The ADP platelet receptor P2Y12 is the target of a family of antithrombotic agents, which includes the thienopyridines (ticlopidine, clopidogrel, and prasugrel) that irreversibly inhibit the receptor and the novel direct and reversible antagonists (ticagrelor, cangrelor, and elinogrel) [108].

A possible role of the P2Y12 receptor in cancer has been shown. Wang et al. [109] demonstrated that tumor metastases are reduced in P2Y12-deficient mice. In animal models of spontaneous or experimentally induced lung metastasis, obtained by injecting Lewis lung carcinoma cells or B16 melanoma cells, respectively, the P2Y12 receptor deficiency was reported to be linked to a reduced weight of metastasis [110]. The coadministration of the antiplatelet drugs aspirin and clopidogrel prevented or delayed the development of hepatocellular carcinoma and improved survival in a mouse model of chronic immune-mediated hepatitis B [110].

Moreover, ticlopidine administration in a rat model of spontaneous pulmonary metastasis of Lewis lung carcinoma suppressed the dissemination process [111]. Ticagrelor reduced the lung colony-forming units and improved survival in an orthotopic 4T1 breast cancer model obtained by inoculating 4T1 mammary carcinoma cells into the mammary pad of female BALB/c mice [112]. In this study, ticagrelor significantly reduced the number of tumor cell-platelet aggregates present in the lung at 10, 30, and 60 min after the intravenous administration of 4T1 cells [112].

Until now, no RCTs assessed the effect of P2Y12 blockage in cancer patients. An epidemiological study recently performed by the analysis of a Spanish primary care database found that clopidogrel use reduced the risk of CRC similar to low-dose aspirin (20–30% reduction in risk), starting at one year of treatment [113]. Interestingly, the dual antiplatelet therapy (aspirin and clopidogrel) had the same effect versus clopidogrel or low-dose aspirin monotherapy [113]. These data support the concept that inhibiting platelet activation signaling pathways is an effective strategy for preventing CRC.

### 6.3. Thrombin Receptor Antagonists

Thrombin induces platelet activation by two PAR family members, PAR-1, the primary thrombin receptor, and PAR-4 [114]. These receptors may play a crucial role in regulating angiogenesis and, in turn, modulate the processes of wound healing and tumor growth [115]. PAR-1 and PAR-4 activation cause the secretion of different sets of a-granules from platelets [116]. Experimental evidence obtained in vitro and in vivo has suggested that the control of PAR1-mediated signaling may represent a promising strategy for malignancy treatment. The subcutaneous injection of MC38 cells, an aggressive C57Bl/6-derived colonic adenocarcinoma cell line, in PAR-1−/− and wild-type mice caused the development of palpable tumors in both genotypes [117]. However, the tumors grew significantly more slowly in PAR-1−/− mice than in control animals [117], suggesting a role for stromal-PAR-1 in tumor outgrowth. Moreover, the PAR-1 antagonist vorapaxar pretreatment of three different epithelial ovarian cancer cells (SKOV-3, OVCAR-3, and CaOV-3) reduced the thrombin-induced cell proliferation, which returned to the baseline values [118].

### 6.4. Glycoprotein IIb/IIIa Antagonists

Glycoprotein IIb/IIIa (GPIIb/IIIa, also known as integrin αIIbβ3), formed via the calcium-dependent association of GPIIb and GPIIIa, is a receptor for fibrinogen and von Willebrand factor [1]. GPIIb/IIIa plays a fundamental role in platelet functions, such as aggregation and adhesion. Much experimental evidence has demonstrated the contribution of platelet receptor GPIIb/IIIa on the crosstalk between platelets and cancer cells [5]. In particular, it was found that melanoma cells (M3Dau) interact with platelets by the platelet receptor GPIIb/IIIa and the GPIIb/IIla-like complex expressed on tumor cells. Additionally, the monoclonal antibody (LYP18) against the platelet GPIIb/IIIa complex inhibited platelet–melanoma cell interactions and platelet–platelet aggregation [119] (Table 1). Lonsdorf et al. [60] found that platelets are relevant for melanoma cell adhesion. They identified an interaction between the integrins GPIIb/IIIa of platelets and ανβ3 of murine B16 melanoma cells. In a mouse model of hematogenous metastasis to the lung, the treatment with mAb blocking the aν subunit of the aνβ3 integrin was associated with a decreased rate of lung metastasis formation by B16 melanoma cells [60].

Some in vitro evidence has shown that tirofiban, abciximab, and eptifibatide (three GPIIb/IIIa blockers currently used in cardiovascular prevention) inhibit cell proliferation and migration and cause apoptosis [120,121,122]. However, the enhanced risk for serious adverse events, such as bleeding, associated with their use (reviewed in [123]) limits their long-term use as chemotherapeutic agents.

## 7. Platelet Omics as a Diagnostic and Prognostic Tool in Cancer

The discovery of platelets occurred in 1881 by Bizzozzero [124], and now, their physiology and role in atherothrombosis are clarified [1,2]. However, the knowledge of novel platelet functions has shown their contribution to cancer development and progression.

Although antiplatelet drugs can be used to prevent CVD and cancer, their administration is associated with side effects, including enhanced risk of bleeding [8]. Some risk factors (i.e., age over 65 years) of enhanced bleeding in long-term antiplatelet treatment after vascular events have been identified [125]. However, it remains to characterize a specific individual phenotype associated with enhanced susceptibility to harm by antiplatelet drug treatments. Furthermore, the patient phenotype receiving increased benefit from the treatment has to be defined. The use of omics technology and big data analysis using artificial intelligence will allow developing a personalized treatment with antiplatelet agents.

Omics analysis can capture dysregulated molecular processes and integrate them into a broader network of biological systems. For example, the advent of omics technology, such as proteomics, metabolomics, and lipidomics, using mass spectrometry, microarray- or RNA-sequencing (RNA-seq)-based transcriptomics, and most recently ribosome footprint-based translatomics has enabled a more detailed understanding of platelet biology. These new analyses will be of valuable help to identify individual fingerprints associated with disease susceptibility and intersubject variability to drug response.

### 7.1. Platelet Proteomics

In 1979, the first proteomic analysis of platelets was reported by Clemetson and colleagues [126]. The most abundant platelet proteins were identified through the two-dimensional gel electrophoresis and Coomassie staining of a platelet protein sample, i.e., actin, myosin, tubulin, fibrinogen, and several glycoproteins [126]. This methodology was applied for the assessment of the proteomic profile in resting [127] and activated platelets [128], platelet releasate [129], platelet MPs [130], and alpha and dense granules [131,132]. Mass spectrometry technology and data analysis have led to a more in-depth quantitative analysis of platelet proteomic profile. For the first time, Burkhart and colleagues reported the quantitative proteomic analysis of resting platelets isolated from healthy volunteers, representing an essential reference for subsequent proteomic studies [133]. In 2013, Wijten et al. [134] published the protein dataset obtained from human activated platelets. They identified 124 proteins released upon activation, whereas Coppinger and colleagues identified more than 300 proteins released by human platelets following thrombin activation [129]. The discrepancy found in proteomic studies regarding the number of proteins identified may be due to the peptide identification criteria, sample prefractionation before mass spectrometry analysis, longer run times, and more sensitive mass spectrometry instruments.

The proteomic repertoire of platelets is derived from (i) megakaryocyte inheritance, (ii) platelet uptake of circulating proteins, and (iii) *de novo* protein synthesis process [135]. Platelets contain all necessary protein synthesis components, such as the precursor messenger RNA (pre-mRNA), a spliceosome to process this pre-mRNA to mature mRNA endoplasmic reticulum, ribosomes, and transfer RNA [136]. Evangelista et al. [135] detected COX-1 mRNA in resting platelets, and after stimulation, *de novo* COX-1 synthesis occurred. This process may interfere with the complete and persistent suppression of TXA_2_ biosynthesis by aspirin necessary for cardioprotection.

A new technology to study platelet translation, based on RNA-seq of ribosome-protected fragments (translatomics), was recently developed [137]. In 2017, Mills and collaborators performed the translatomics of thrombin-stimulated platelets [138]. Interestingly, they found a significant, but moderate, correlation between platelet transcriptome with the platelet translatome and the platelet translatome with the proteome. This data reinforced the concept that the platelet protein synthesis participates only marginally in platelet proteome composition [139].

The platelet proteome may be considered both a diagnostic and predictive biomarker in cancer patients. Platelets store high levels of growth factors, such as VEGF, which is uptaken mainly from the circulation [38]. In cancer patients, VEGF is sequestered by platelets, and the total quantity of VEGF is 6.5 times higher than that in serum and 28.2 times higher than that in plasma [139]. Further studies are needed to clarify how platelets contribute to the increase of VEGF in cancer and whether the amount of VEGF sequestration by platelets could influence the response to anti-VEGF therapy.

Several human studies performed in cancer patients revealed elevated levels of circulating angiogenesis-regulator proteins versus healthy subjects, such as VEGF, ANGPT-1, MMP-2, platelet factor 4 (PF-4), and PDGF [140], which can be uptaken by platelets. Thus, fluctuations in the platelet proteome may serve as surrogate markers of tumor activity. Salgado et al. [141] have shown that platelet VEGF levels positively correlated with tumor growth kinetics in breast cancer patients. In patients with colorectal cancer, Peterson et al. [142] found that the platelet concentrations of different angiogenesis regulatory proteins, i.e., VEGF, PDGF, and PF4, were higher than healthy controls. Using these data, they accurately discriminated between patients with cancer from the control group [142]. More recently, Sabrkhany et al. [143] performed proteome analysis in platelets isolated from early-stage lung or head of pancreas cancer and healthy matched-controls. In this study, the authors identified 4384 proteins, 85 significantly modulated in early-stage cancer than controls, and seven associated with early-stage cancer [143].

### 7.2. Platelet Transcriptome

Transcriptomics analysis consists of studying the entire landscape of RNA transcripts in a cell type or tissue. In addition to the transcriptome studies on coding mRNAs, the noncoding transcriptome (microRNAs, YRNAs, circular RNAs, long noncoding RNAs) is also gaining attention for its role as platelet function marker in various conditions [144,145]. Several studies have shown the association between the transcriptomic profile of platelets with different pathological conditions, such as inflammatory [146], hematological [147], oncologic [148], autoimmune [149], metabolic [150], nephrological [151], and cardiovascular diseases. In particular, distinct platelet transcriptomes for patients with stable and acute coronary heart disease [152,153,154], coronary artery bypass graft surgery [155], peripheral artery disease [156], and atrial fibrillation [157] were described.

The platelet transcriptome analysis is of particular interest since it can define platelet-educated platelets and their use as a diagnostic and prognostic surrogate in different types of tumors.

## 8. Tumor-Educated Platelets as a Liquid Biopsy Tool in Cancer

Several studies have shown that the presence of tumor disease influences the platelet count, volume, activation status, proteins, and RNA content, and these changes occurred already at the early stages of the disease [33].

Liquid biopsy represents an important tool in cancer for its diagnostic and prognostic values. It is a minimally invasive method for detecting and monitoring cancer. Liquid biopsies permit the early detection of cancer and prognosis for the individual patient for tumor stage and identification of new targets for personalized treatment. However, its clinical use requires validation in large-size clinical studies with outcomes. Until now, several blood-based biosources are evaluated as liquid biopsies: the analysis of cancer cell-free DNA and RNA, proteins, and circulating tumor cells [39,158,159,160]. Furthermore, EVs (exosomes) have been suggested as liquid biopsy since they harbor proteins and RNA molecules and surface membrane proteins correlated to organ tropism for cancer metastasis [161].

The first observation of the significant differences of the platelet genomic profiles in cancer patients versus healthy individuals was reported by Calverley et al. [162]. They found that 197 platelet-associated genes were downregulated in patients with metastatic lung cancer, and multiple genes were also spliced differentially between the control and patient groups [162]. Moreover, Nilsson et al. [163] reported that platelets from cancer patients could actively absorb tumor-derived EVs and take up RNA from tumor cells. For the first time, Best et al. [148] defined that the analysis of tumor-educated platelets (TEPs) can represent a potential tool for cancer diagnosis and screening. TEPs from cancer patients can locally and systemically release EVs, which can deliver their cargo, including RNAs [163,164,165] and proteins [166]. Additionally, TEPs can alter their spliced RNA profile [39,149,162,167]. The analysis of the transcriptome of TEPs from cancer patients is a potential diagnostic tool for several types of cancer [39]. TEPs were evaluated in a study performed in patients with glioma [163]. It was shown that cancer cells transfer mutant RNA into blood platelets and TEPs isolated from glioma patients contained EGFRvIII mutant RNA molecules [163]. Similar evidence was found in platelets from non-small-cell lung carcinoma (NSCLC) or prostate cancer (PC) patients [164,165]. In NSCLC patients, the EML4-ALK rearrangement was found in platelets [164]. PC platelets contained the transcripts for the PC-associated biomarkers, such as kallikrein-related peptidase-2 and -3, folate hydrolase 1, and neuropeptide-Y [166]. Using an algorithm, the swarm-intelligence, to optimize the biomarker spliced RNA panel, it was possible to diagnose the late-stage NSCLC with great accuracy [167]. The recent analysis in patients with multiple myeloma identified differential platelet-RNA profiles that discriminated between healthy individuals and patients with smoldering multiple myeloma [39]. Best et al. [148] developed the thromboSeq platform, in which the use of RNA sequencing methodology identified the spliced RNA profiles to discriminate patients with localized and metastasized cancer from healthy individuals with 84% to 96% accuracy, respectively. This technology can permit defining the organ-of-origin of the primary tumor with 71–89% accuracy [148]. The spliced RNA profile of NSCLC patient platelets identified the presence of tumor tissue-derived molecular biomarkers, such as the EGFR and KRAS mutations and HER2 and MET amplification with 85% to 95% accuracy [148]. The gene ontology analysis of RNA profiles found in platelets revealed that these RNAs are implicated in platelet activity, platelet vesiculation, and a decrease in RNA maintenance and splicing was also observed [148]. These analysis results were corroborated by another study performed in NSCLC patients, which showed that the platelet-RNA panel containing 698 RNAs overlapped by 77% with an 1820-RNA gene panel associated with younger platelets, including the RNA for membrane marker P-selectin, previously correlated to younger reticulated platelets [166].

Interestingly, Clancy et al. [168] proposed a specific RNA profile that discriminates between small (old) and large (young) platelets. In large platelets, an enrichment for gene ontologies associated with platelets/vesicles and hemostasis was found. Gene ontology studies on tumor-educated platelets suggest that cancer patients are characterized by younger platelets, possibly contributing to their enhanced thrombotic potential in cancer [168]. It has been proposed that the older, smaller platelets accumulate a repertoire of vascular cell-derived RNAs during their lifespan [168], indicating that platelet subpopulations correlate with enrichment or depletion of specific transcripts.

Despite the great interest in the platelet molecule content analysis, it is necessary to validate how to collect and isolate platelets and EVs and the omics assessment within different platforms before recommending it as a diagnostic and prognostic tool. An international collaboration network of experts should be organized to discuss and define the validated recommendations on this complex field.

## 9. Platelet as a Drug Delivery Tool in Cancer

Different cancer types can be successfully treated and even cured if treatments, including radiation, chemotherapy, immunotherapy, or surgery, are performed promptly following early diagnosis. All these approaches aim to contain tumor growth or prevent metastatic formation. Chemotherapy, which uses anticancer drugs, represents one of the most common, standardized, and known therapeutic choices. However, chemotherapeutic agents have several limitations, such as the lack of selectivity, the development of severe side effects, limited efficacy, and drug resistance occurrence [169]. Moreover, the metastatic process, which represents the primary cause of cancer-associated mortality, is particularly challenging to be constrained or abolished by the traditional therapeutic approaches [170]. Thus, the need for novel strategies has opened new avenues, among which natural cell-based drug delivery has recently received considerable attention [171].

Several types of mammalian cells, including mesenchymal stem cells, red blood cells, macrophages, neutrophils, and T cells [172,173,174,175,176], have been tested as ideal carriers for drug delivery to potentiate the therapeutic efficacy minimizing toxic and side effects.

In this view, more recently, platelets have been exploited as carriers for cancer therapy, given their recognized role in the complex, bidirectional interaction with malignant cells either in the blood or in the tumor microenvironment [16,31,123,177,178].

Besides the inherent tropism of platelets to vascular injury [179], particular interest for the drug delivery process is a recent finding relative to extravasated platelet presence in the microenvironment of different kinds of tumors, including pancreatic cancer, hepatocellular carcinoma, gastric, and CRC [180]. In this peculiar environment, platelets interact with cancer cells without giving rise to the aggregation process. The mechanism underlining platelet extravasation and its role in tumor progression and the metastatic process is still poorly understood. A role has been ascribed to P-selectin, as shown by Qi and coworkers, who demonstrated that P-selectin deficiency abolishes platelet deposition within solid tumors and platelet focal adhesion kinase (FAK) protein [181]. In in vivo experiments using orthotopic models of ovarian cancer, Haemmerle et al. demonstrated that the administration of the FAK-inhibitor GSK2256098 was able to reduce platelet infiltration and to prevent tumor growth after the withdrawal of the antiangiogenetic drugs bevacizumab or pazopanib [182]. The reported homing efficacy of platelets toward tumor cells is another critical characteristic that makes them a promising tool as drug delivery vehicles for anticancer agents.

Additional features make these anucleate circulating cells particularly attractive as a novel therapeutic cell-based strategy. Indeed, they: (i) present a relatively long lifespan in circulation (8–10 days), which could improve the pharmacokinetics of drugs usually administered intravenously; (ii) can be easily harvested from the patient with minimally invasive procedures and without ethical concerns, (iii) can be engineered in vitro and reinfused to the patient, (iv) can be targeted with nanoparticles and even with cells directly into the bloodstream thanks to the presence of specific surface binding sites such as the highly expressed GPIIb/IIIa [183]. A bispecific tandem single-chain antibody (Tand-scFvSca-1+GPIIb/IIIa) able to bind to both activated platelets via GPIIb/IIIa receptor and to a subset of peripheral blood mononuclear cells (PBMCs) expressing the stem cell antigen-1 (Sca-1) receptor has recently been developed [183]. When the targeted PBMCs were injected into a mouse model of myocardial infarction, their homing capability was increased and marked beneficial effects were found: decreased fibrosis, increased capillary density, and restored cardiac function. This innovative platelet-based cell-targeted therapy might have a relevant translational interest. Indeed, human CD34 might be equivalent to the murine Sca-1. CD34 is highly expressed in different types of promising regenerative cells. Thus, its use in humans may allow the development of platelet-based targeted cell therapies similar to those obtained with Sca-1 in mice.

### 9.1. Platelet Loading

Platelets are also known to act as scavengers in the bloodstream where they take up small molecules and nucleic acids and particulate material, viruses, and bacteria [184,185]. Unlike a phagocytic process in which the engulfed material is metabolized inside the cells [186], it remains intact in platelets. This is why platelets are defined as covercytes, not phagocytes. The molecules can flow simply into the platelet; however, platelets can evaginate the open canalicular system, enhancing their surface [187].

**Figure 1:**
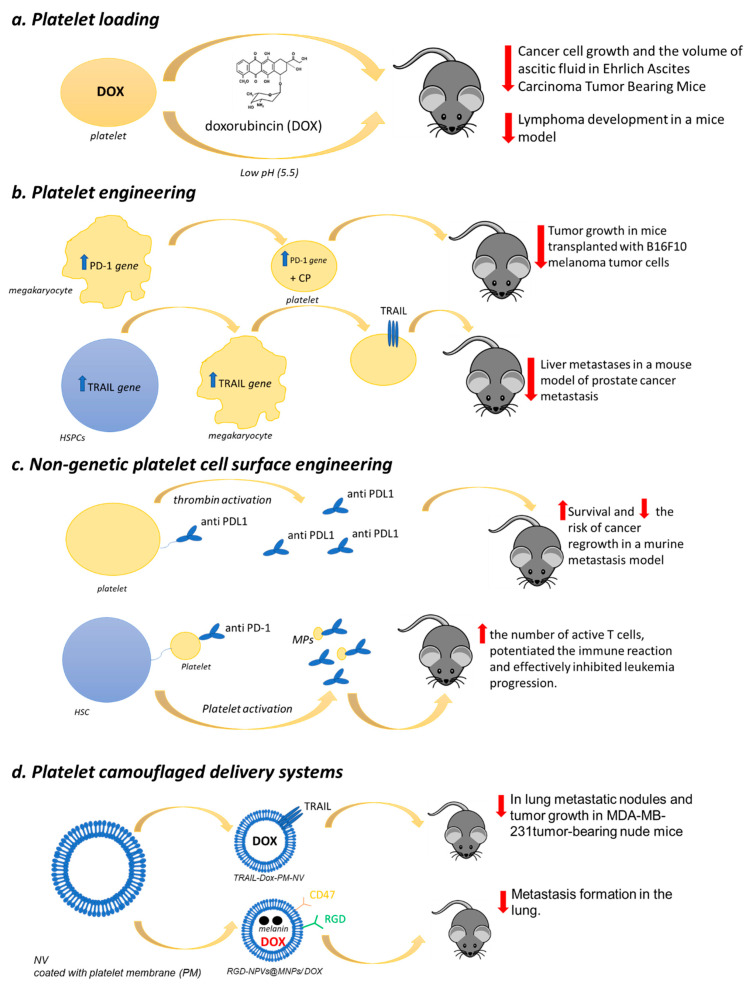
**Platelet-based drug delivery systems.** Four different types are categorized: (**a**) Platelet loading; (**b**) platelet engineering; (**c**) nongenetic platelet cell surface engineering; (**d**) platelet camouflaged delivery systems. Their antitumoral effects in in vivo models are also reported.

Sarkar et al. [188] demonstrated that platelets loaded with doxorubicin (DOX), an anticancer drug that inhibits topoisomerase II, were able to inhibit cancer cell growth and significantly reduce the volume of ascitic fluid in Ehrlich Ascites Carcinoma (EAC) tumor-bearing mice (Figure 1). Interestingly, thanks to platelet target specificity toward cancer cells, the therapeutic effect was achieved by a drug dose lower than that usually prescribed, thus reducing toxicity.

More recently, DOX was loaded into platelets through the open canalicular system and was reported to effectively treat lymphoma in a mice model obtained by subcutaneous injection of Raji cells (Burkitt’s Lymphoma; B lymphocyte cell line) [189]. The drug was efficiently loaded into platelets with minimal influence on their morphology and functionality and was stable. The DOX release from DOX-platelets was pH-dependent (the maximal rate was at pH 5.5, whereas it was much lower at pH 7.4) (Figure 1). This result is of particular interest given that, in tumor tissue environments, and even more in cancer cells, pH values are less than 7. Tumor-bearing mice treated with DOX-platelets showed a significant reduction in the tumor size compared with both controls and free DOX-treated animals suggesting a more significant therapeutic efficacy of the drug delivery system. Bodyweight loss, considered an early side effect, was reported only in animals injected with the free drug. Moreover, myocardial injury, the most severe DOX-induced side effect, was not observed in the group treated with the DOX-platelet system, indicating a reduction in drug toxicity.

### 9.2. Platelet Engineering

In recent years, the genetic engineering of blood cells has been explored, demonstrating promising drug delivery carriers based on their biocompatibility, circulation performance, and targetability [190].

In particular, there is a growing interest in exploring strategies for engineering cell surfaces to manipulate cell–cell and cell–extracellular matrix interactions. This approach has led to the approval by the Food and Drug Administration (FDA) of autologous T cells engineered to express a chimeric antigen receptor (CAR) as first “living drugs” for the treatment of refractory pre-B cell acute lymphoblastic leukemia and diffuse large B cell lymphoma [191].

Genetic engineering is an approach that cannot be directly applied to platelets given their peculiar feature of anucleate and terminally differentiated cells. Thus, much effort has been put into the genetic engineering of platelet precursor cells, i.e., megakaryocytes (MK).

The first studies were carried out mainly for targeted delivery of therapeutic agents at sites of vascular injury or the correction of genetic defects tested in animal models of Glanzmann Thrombasthenia, Bernand–Soulier Syndrome, or Hemophilia A [192,193,194,195]. However, the transfection of primary MK has proved to be a challenging process given the fragile nature of these cells and the difficulty of obtaining a sufficient cell number (in the bone marrow, they represent approximately 0.4% of the total nucleated cells) [196].

A strategy to overcome these difficulties was to manipulate progenitor or precursor cells to generate platelets for specific clinical applications [197].

Substantial progress in cancer treatment is due to the development of immune checkpoint inhibitors (ICI) such as an anticytotoxic T-lymphocyte antigen 4 (CTLA4) monoclonal antibodies (mAb) (ipilimumab) and anti-programmed-death-1 (PD-1) mAbs (nivolumab and pembrolizumab), able to overcome the tumor-induced immune escape [198,199,200]. This pharmacological approach has led to long-term survival in patients with several kinds of cancer, including melanoma, cervical, breast, and non-small-cell lung cancer [201,202,203,204], and its use is increasing in clinical practice. Unfortunately, the ICI efficacy is observed in a small proportion of treated patients, and drug resistance development often occurs.

Zhang and coworkers [205] recently generated genetically engineered murine MK progenitor cells able to give rise to mature platelets expressing PD-1, which were also loaded with cyclophosphamide, a regulatory T cells (Treg) inhibitor [206] (Figure 1).

These modified platelets were administered in mice transplanted with B16F10 melanoma tumor cells. Subsequently, the animals were subjected to partial surgery to mimic residual tumors in the surgical bed. The PD-1-platelets were able to accumulate within the tumor microenvironment and to block the PDL-1, thus inhibiting the activity of the immune-suppressive Treg and favoring the activity of CD8+ T lymphocytes.

Moreover, the targeted release of their cyclophosphamide cargo potentiated the anticancer effects of CD8+ T lymphocytes (Figure 1). Thus, PD-platelets infusion significantly reduced and delayed the tumor growth, whereas the infusion of wild-type platelets or PBS did not.

The PD-1-platelet treated mice also displayed a longer lifespan, surviving more than 60 days without showing signs of toxicity.

For anticancer therapy, platelets were also engineered to express surface-bound necrosis-factor-related apoptosis-inducing ligand (TRAIL), a cytokine that induces apoptosis selectively in a wide range of cancer cells while sparing normal ones [207] (Figure 1).

Murine C57BL6-derived hematopoietic stem and progenitor cells (HSPCs) transduced with the lentiviral transgene and differentiated into platelets in vitro were used [208]. The insertion of the αIIβ promoter in TRAIL cDNA of the genome of HSPCs was used to control TRAIL expression in platelet lineages following HSPC differentiation. The TRAIL-expressing platelets were able to kill cancer cells in vitro. The engineered HSPCs were transplanted into NOD SCID gamma (NSG) bone marrow to guarantee TRAIL-expressing platelet continual production in circulation (Figure 1). More interestingly, this drug delivery system significantly reduced liver metastases in a mouse model of prostate cancer metastasis compared with control mice receiving empty vector-transduced HSPCs. Additionally, this approach could represent a strategy to overcome the repeated administration problem for chemotherapeutic agents.

A novel approach to trigger the synthesis of therapeutic proteins in platelets without the need for precursor cell engineering comes from Chan and colleagues [209]. They developed a system aimed at synthesizing RNA and proteins encapsulated in liposomes and called protocells. Platelets were able to take up the protocells as assessed by flow cytometry and confocal microscopy and, upon light control activation, a dramatic increase in platelet mRNA was observed. This represents a proof-of-concept that it is possible to induce a controlled transcription of exogenous RNA in anucleate cells, opening new avenues in the platelet-based therapeutic approach.

### 9.3. Nongenetic Platelet Cell Surface Engineering

Platelets can also be directly modified through nongenetic cell surface engineering to act as drug delivery systems thanks to the abundance of different kinds of binding sites on their plasma membrane. Monoclonal antibodies against programmed-death ligand 1 (aPDL1) were recently covalently linked to resting platelets via a bifunctional maleimide linker [210] (Figure 1). The complex (aPDL1-platelets) resulted in being stable, and aPDL1 was promptly released from the platelet surface upon thrombin activation. Once injected into mice models of experimental metastasis and cancer recurrence, aPDL1-platelets effectively prolonged animal survival by reducing the risk of cancer regrowth and metastatic dissemination.

In order to ameliorate the targetability of platelets as a drug delivery tool for the treatment of leukemia, Hu et al. [211] engineered platelets to express anti-PD-1 antibodies and subsequently decorated their surface with hematopoietic stem cells (HSCs) through a click reaction, which takes place in a water solution with a pH range of 6–8 and at a temperature of 37 °C without generating toxic byproducts [212] (Figure 1). This method resulted in a lack of the aggregation of platelet–HSC assembly and the preservation of platelet function. The generated innovative drug delivery system determined a significantly increased efficacy of the checkpoint blockade therapy by taking advantage of the recognized homing capability of HSC, which drove platelets toward bone marrow. The treatment increased the number of active T cells, potentiated the immune reaction, and effectively inhibited leukemia progression. Moreover, it also resulted in protection against a subsequent challenge with leukemia cells.

### 9.4. Platelet-Camouflaged Delivery Systems

In the last decade, growing interest has emerged in applying nanotechnology to cancer, and different kinds of therapeutic nanoparticle (NP)-based drug delivery systems, including liposomes, polymeric, and albumin NPs have been proposed for approval in cancer treatment or are in the advanced experimental phase [213]. However, conventional NP-based systems present some critical issues in biodistribution and targetability so that after intravenous injection, they do not accumulate at the tumor site in a sufficient amount [214].

Nanoparticle characteristics such as surface features, composition, porosity, elasticity size, and geometry can influence these biological processes impairing cancer treatment efficacy.

Cell-membrane-coated NPs (CMCNPs) have been developed as a novel pharmacological strategy. This “Trojan horse” technology allows NPs to increase their circulation time and reach the tumor cells [215].

Different kinds of CMCNPs have been developed using various cells, including erythrocytes, mesenchymal stem cells, white blood cells, red blood cells, platelets, cancer cells, and bacteria [216], and most of them are in the preclinical development stage.

A CMCNP has recently been developed by using platelet membranes. The biomimetic platform was designed for site-specific and sequential delivery of two anticancer agents: TNF-related apoptosis-inducing ligand (TRAIL), known to induce cancer cell apoptosis by selectively interacting with the surface death receptors (DR4, DR5) [211], and DOX (TRAIL-Dox-PM-NV) [216] (Figure 1). When injected into MDA-MB-231 tumor-bearing nude mice, this delivery system significantly inhibited tumor growth. The histologic analysis results showed a remarkable cancer cell remission in animals treated with TRAIL-Dox-PM-NV compared with controls confirmed by situ TUNEL assay, which revealed the highest apoptosis in mice’s tumors treated with this biomimetic drug delivery platform. Moreover, the hematoxylin and eosin staining showed that in mice treated with TRAIL-Dox-PM-NV, a significant reduction in metastatic lung nodules was achieved compared with animals treated with saline or TRAIL-Dox-NV, thus supporting, for the first time, the hypothesis that nanoparticles camouflaged with platelet membrane can have potential application in anticancer therapy.

Increasing evidence has been accumulating on the efficacy of platelet membrane coated delivery systems. The presence of surface marker proteins such as P-selectin, CD47, and CD44 receptors on the platelet membranes protect from immunogenic clearance and favor the specific adhesion to injured tumor tissues of these camouflaged delivery platforms.

Jing and coworkers developed a platelet-camouflaged nanococktail by encapsulating melanin nanoparticles (MNPs) and DOX inside RGD peptide (c(RGDyC))-modified nanoscale platelet vesicles (RGD-NPVs@MNPs/DOX) [217] (Figure 1). The biocompatible system was tested in a tumor model generated by injecting nude mice with 5 × 105 DOX-resistant breast cancer (MDA-MB-231/ADR) and being very effective in suppressing tumor growth.

It is noteworthy that RGD-NPVs@MNPs/DOX combined with laser (808 nm, 1.5 W, and 10 min) improves the chemophotothermal elimination of resistant cells and tumor vasculature, thus, completely inhibiting metastasis formation in the lung.

Platelet membrane-coated NP delivery has been tested for its ability as a radiotherapy enhancer [218]. This is a very relevant clinical issue, given that radiotherapy is still one of the most efficient treatments for solid tumors. A mesoporous silica-coated bismuth nanorod (BMSNR) was camouflaged by a platelet membrane (BMSNR@PM). The biomimetic system was injected into BALB/c mice bearing 4T1 tumors, as a model of breast cancer, followed by near-infrared irradiation (1 W cm^−2^, 10 min) (NIR) and by local low-dose X-ray irradiation (IR) (5 Gy, 1.0 Gy min^−1^) (BMSNR@PM/NIR/IR). BMSNR@PM/NIR/IR caused a significant reduction in tumor mass and volume, confirming this NP-camouflaged system’s capability to induce a synergistic therapeutic activity and benefit greater than phototherapy radiotherapy given alone.

A similar effect has been reported for a platelet coated delivery system in potentiating the therapeutic outcome of cancer immunotherapy by ferroptosis, a recently recognized iron- and reactive-oxygen-species-dependent form of regulated cell death [219].

Magnetic nanoparticles (Fe_3_O_4_) were loaded with Sulfasalazine (SAS), a drug primarily used in the treatment of rheumatoid arthritis and ulcerative colitis [220], which was also reported to suppress tumor growth and induce ferroptosis [221]. Magnetic nanoparticles loaded with SAS were camouflaged by platelet membranes (Fe_3_O_4_-SAS@PLT) and injected into 4T1 metastatic tumor-bearing mice alone or in combination with anti-PD-1 antibody after lung metastasis development. Their ability to target the metastatic lung lesions was monitored by in vivo imaging technique. The biomimetic system significantly enhanced the antitumor immune response, causing an almost complete suppression of tumor metastasis. Moreover, tandem mass tags (TMT)-based proteomic analysis showed that Fe_3_O_4_-SAS@PLT treatment was able to induce M2 phenotype macrophage polarization toward the M1 phenotype with recognized antitumor properties.

Platelet-based biomimetic formulations need to be optimized for use in cancer treatments. Several issues remain to be addressed: (i) platelets have to be isolated and functionalized in vitro before being reinjected with a risk of contamination, (ii) platelets contribute to thrombosis and tumor development. Thus, their administration might also exacerbate pathological conditions, (iii) platelets are highly reactive, and their unpredicted activation could cause drug-induced toxicity or reduced efficacy. The use of platelet membranes to obtain camouflaged drug delivery systems can overcome these critical aspects. However, the integrity of both platelet membranes and NP core integrity and functionality in the bloodstream is not easy to characterize. Despite evidence of safety for platelet membrane camouflaged NPs, there is no information on their biocompatibility and lack of toxicity in long-term treatments in vivo, and further careful investigations are still required.

## 10. Conclusive Remarks

Numerous pieces of evidence sustain the novel roles of platelets beyond hemostasis and thrombosis. Platelets are rapidly activated in response to vascular and epithelial damage and trigger numerous signaling, translating into an acute inflammatory response to repair the cellular damage. However, uncontrolled platelet activation contributes to chronic inflammation development, considered a hallmark of atherothrombosis and tissue fibrosis and cancer. The role of platelets in tumor metastasis is now fully appreciated, and the use of antiplatelet agents should be added to adjuvant antitumor therapies. However, numerous questions remain to be addressed, significantly associated with developing a personalized use of aspirin and other antiplatelet agents to improve anticancer efficacy and reduce enhanced bleeding associated with their use. The progress of omics technology associated with available bioinformatics tools will help identify individuals susceptible to cancer development (before any clinical symptoms detectable) and direct the appropriate anticancer therapy. The detailed analysis of platelet and EV content will help clarify patient features. This information will contribute enormously to early cancer diagnosis, monitor drug treatments, and define patient prognosis. Therefore, the following years will be exciting because the tremendous research efforts will lead to significant advances in cancer diagnosis and treatments.

The evidence of platelet role in tumor growth and metastasis has substantially increased since the first experimental in vivo findings by Gasic et al. [222]. Platelets can play a relevant function in primary tumor growth and all metastatic dissemination steps [4]. They can infiltrate the tumor microenvironment and directly interact with cancer cells, thus modifying their phenotype toward a more malignant type [5,83]. Additionally, platelets interact with circulating tumor cells in the bloodstream, thus preventing the deadly attack of the immune system and promoting the adhesion to the endothelium, providing signals to establish a premetastatic niche [4]. The crosstalk between platelets and cancer cells accounts for the release of several soluble mediators and MPs by platelets [5,31,74]. Platelet-derived MPs can promote cell malignancy changes by delivering genomic (i.e., RNAs, miRNAs) and protein factors.

Besides the release of biologically active mediators, platelets can uptake and store many circulating molecules [166]. This peculiarity of platelet biology allows the development of novel biomarkers of disease status based on platelet signatures. Liquid biopsy is a minimally invasive tool helpful for cancer detection and disease or treatment monitoring. Until now, several in vivo studies have been performed to characterize the transcriptomic of TEPs and propose it as a liquid biopsy for cancer detection [148,167]. This methodological approach may offer advantages over other blood-based tests since platelets represent the most abundant cells in the blood, with high-quality RNA and the capacity to change their genomic and proteomic profile in response to the different pathological conditions, including cancer [39]. Further clinical validation studies are needed to progress toward blood-based screening programs for early cancer detection.

Platelet capacity to specifically interact with tumor cells suggests that platelets can act as a biomimetic drug delivery system for antitumor therapy [223,224]. Indeed, the rapidly developing field of cell-based drug carrier systems indicates platelets as smart candidates for anticancer agent delivery due to their peculiar features: (i) simple separation from blood and ready availability, (ii) long circulation time, (iii) natural selectivity toward damaged tissue and tumor microenvironment, and (iv) weak antigenicity and immunogenicity [41]. Although growing evidence suggests translational feasibility and clinical value for in the vivo application of platelet-based biomimetic formulations, they remain to be optimized and investigated further for use in cancer treatments.

## Figures and Tables

**Table 1:** Molecular determinants involved in the interaction between platelets and cancer cells and their blockage by pharmacological tools.

Platelet Constituent	Cancer Cell Constituent	Cells Responses	Pharmacological Tools	References
Integrin αIIbβ3 (or GPIIb/IIIa)	Integrin αvβ3	Cancer cell capacity to adhere to the endothelium was increased by platelet activation under flow	Abciximab	Lonsdorf et al., 2012; Felding-Habermann et al., 1996 [60,61]
Integrin αIIbβ3 (or GPIIb/IIIa)	?	Platelet receptor FcɣRIIa activation and ADP release induced platelet aggregation	Abciximab	Mitrugno et al., 2014 [62]
Integrin αIIbβ3 (or GPIIb/IIIa)	GPIIb/IIa-like complex	ADP released from cancer cells induces platelet aggregation, degranulation, and the formation of platelet–tumor cell aggregates	mAb LYP18 against GPIIb/IIIa	Boukerche et al., 1995 [63]
Integrin α6β1	ADAM9	Induction of platelet activation, granule secretion, and subsequent endothelial transmigration of tumor cells	GoH3 (Integrin α6-blocking antibody)	Mammadova-Bach et al., 2016 [64]
P-selectin	Mucin-type glycoprotein	Direct interaction between cancer cells and activated platelets	Crizanlizumab	Mannori et al., 1995; Man et al., 2020 [65,66]
P-selectin	PSGL-1	Induction of platelet activation and platelet/cancer cell aggregates	Crizanlizumab	Man et al., 2020; Gong et al., 2012 [66,67]
P-selectin	CDC44	Induction of platelet/cancer cell aggregates under shear stress	Crizanlizumab	Man et al., 2020; Alves et al., 2008 [66,68]
P-selectin	PCLP1	Induction of platelet activation and formation of platelet/cancer cell aggregates	Crizanlizumab	Man et al., 2020; Larrucea et al., 2007 [66,69]
GPVI	Galectin-3	COX-2 overexpression and EMT induction in cancer cells	Revacept	Dovizio et al., 2013 [17]
CLEC-2	Podoplanin	Platelet aggregation and induction of platelet/cancer cell aggregates	mAb NZ-1 against CLEC-2	Suzuki-Inoue et al., 2007; Chang et al., 2015; Kato et al., 2006; [70,71,72]

**Table 2:** Randomized clinical trials that compared the effect of aspirin in the prevention of colorectal adenoma or cancer versus comparator.

Treatment	Patients	Primary End-Point	Results (95%CI)	References
Placebo or 325 mg of aspirin each day for 5 years	Male physicians aged between 40 and 84 years	Incidence of total cancer	RR: 1.15 (0.80–1.65) for colorectal cancer	Gann et al., 1993. [91]
Placebo or aspirin 100 mg of aspirin every other day for an average of 10.1 years	Healthy women aged at least 45 years	Confirmed newly diagnosed invasive cancer at any site	RR: 1.01 (0.94–1.08) for total cancer; RR: 0.97 (0.77–1.24) for colorectal cancer	Cook et al., 2005 [92]
Placebo or 81 mg or 325 mg of aspirin daily for 2.8 years	Patients with a recent history of histologically documented (removed) adenomas	Proportion of patients in whom one or more colorectal adenomas were detected	Any adenoma; RR: 0.81 (0.69–0.96) for ASA 81 mg, *p* = 0.04; RR: 0.96 (0.81–1.13) for ASA 325 mg Advanced lesion; RR: 0.59 (0.38–0.92) for ASA 81 mg; RR: 0.83 (0.55–1.23) for ASA 325 mg	Baron et al., 2003. [93]
Placebo or 325 mg daily of enteric-coated aspirin for 2.6 years	Patients who had histologically documented colon or rectal cancer with a low risk of disease recurrence	Detection of adenomas in the large bowel by either colonoscopy or sigmoidoscopy after randomization	RR: 0.65 (0.46–0.91) *p* = 0.004	Sandler et al., 2003. [94]
Placebo or soluble aspirin (160 or 300 mg daily) for 1 year	Patients with a history of colorectal adenomas	Adenoma recurrence after 1 year	RR: 0.73 (0.52–1.04) *p* = 0.04 for both doses	Benamouzig et al., 2003 (APACC trial) [95]
Placebo or soluble aspirin (160 or 300 mg daily) for 4 years	Patients with a history of colorectal adenomas	Adenoma recurrence after 4 years	RR: 0.96 (0.75–1.22) for both doses	Benamouzig et al., 2012 (APACC trial) [96]
Aspirin (300 mg daily) versus folate supplements (0.5 mg/d) for about 2.6 years	Patients with an adenoma removed in the 6 months before recruitment	A colorectal adenoma diagnosed after baseline	RR: 0.79 (0.63–0.99) *p* = 0.04	Logan et al., 2008. [97]
Aspirin (600 mg/d) and/or resistant starch (30 g/d) for 17 years	FAP young patients	Polyp number in the rectum and sigmoid colon	RR: 0.77 (0.54–1.10)	Burn et al., 2011 (CAPP1 trial) [98]
Aspirin (600 mg/d) and/or resistant starch (30 g/d) or placebo for a mean of 10 years	Lynch syndrome (hereditary nonpolyposis colon cancer or HNPCC)	Development of colorectal cancer	HR: 0.65 (0.43–0.97; *p* = 0.035) for aspirin vs. placebo	Burn et al., 2020 (CAPP2 trial). [100]
81 or 100 mg of aspirin daily for 10.7 years	Type 2 diabetic patients	Time to first cancer incidence	HR: 0.92 (0.73–1.14) *p* = 0.4; adjusted HR, 0.66 (0.43–0.99) *p* = 0.04	Okada et al., 2018. [101]
100 mg of aspirin or placebo for 7.4 years	Subjects with a diagnosis of diabetes mellitus (any type) aged >40 years	First serious vascular event	RR: 0.98 (0.84–1.15) for cancer-related mortality	Bowman et al., 2018 (ASCEND study) [102]
100 mg of aspirin or placebo for 4.7 years	Healthy Elderly (>70 years)	Composite of death, dementia, or persistent physical disability	HR: 1.35 (1.13 to 1.61) for cancer mortality; HR: 1.04 (0.95–1.14) for cancer incidence	McNeil et al., 2018; McNeil et al., 2020. [103,104]
100 mg of aspirin or 300 mg or placebo daily for at least five years	Patients who have undergone potentially curative treatment for breast, colorectal, gastroesophageal, or prostate cancer	Invasive disease-free survival for the breast cohort, disease-free survival for the colorectal cohort, overall survival for the gastroesophageal cohort	Ongoing	Coyle et al., 2016 (ADD-ASPIRIN trial) [105]

RR: relative risk; HR: hazard ratio. Association pour la Prévention par l’Aspirine du Cancer Colorectal (APACC); Colorectal Adenoma/Carcinoma Prevention Programme (CAPP); A Study of Cardiovascular Events in Diabetes (ASCEND).
